# P10-01 Acceptability of videoconferencing physical activity intervention among elderly in rural areas: qualitative study

**DOI:** 10.1093/eurpub/ckac095.140

**Published:** 2022-08-29

**Authors:** Meggy Hayotte, Pierre Thérouanne, Anne Vuillemin, Aurélie Van Hoye, Fabienne d'Arripe-Longueville, Laure-Anne Seytor

**Affiliations:** 1 Université Côte d'Azur, LAMHESS, France; 2 Laboratoire Anthropologie, Psychologie Cliniques, Cognitives et Sociales (LAPCOS), Université Côte d'Azur, Nice, France; 3 Laboratoire Approches psychologiques et épidémiologiques des maladies chroniques (APEMAC), Université de Lorraine, Nancy, France

**Keywords:** Elderly, Physical activity, Technology acceptability, eHealth, Videoconferencing

## Abstract

**Background:**

Elderly's sustainable engagement in an active lifestyle is a complex phenomenon, implying a plurality of physical, psychological and environmental barriers and facilitators. One of the key factors limiting physical activity (PA) in rural area is the lack of accessibility to PA offers. The use of videoconferencing is a promising solution in terms of acceptability and efficiency to promote PA. However, the acceptability (initial stage of the technology adoption process) of the videoconferencing is still underexplored, as well as its role in promoting PA in rural areas. The purpose of this study was to explore, through the perceptions of the elderly and policymakers, the mechanisms of acceptability of the development of PA interventions using videoconferencing for elderly living in rural areas.

**Methods:**

Elderly (n = 10) and policymakers (n = 12) from 11 different rural town of the South of France participated in the study. Semi-structured individual interviews were conducted, where a video presentation of PA interventions using videoconferencing was played as support. The interview guide assessed the acceptability of this type of intervention through the following themes: (1) perceptions of benefits, (2) barriers, and (3) intentions of use.

**Results:**

Preliminary results showed shared perceptions between elderly and policymakers for barriers (e.g., lack of real social ties, lack of technological equipment) and benefits (e.g., breaking loneliness, overcome weather conditions). However, intentions of use are heterogeneous according to individual characteristics (e.g., “young” elderly between 60 to 65 years are more favourable to the use of technologies) and the specificity of towns (e.g., in the smallest towns with less than 500 people, elderly are less inclined to change their habits).

**Conclusions:**

This study showed that the development of videoconferencing PA intervention could be accepted by elderly living in rural areas, taking into account some conditions (e.g., offer as punctual and non-regular use, set up individual support for the use of technology). Additional quantitative studies should be conducted for a more comprehensive understanding of the factors influencing the videoconferencing PA intervention acceptability in rural areas.

